# 2-(1-Adamant­yl)-1-{4-[(2-chloro-9-isopropyl-9*H*-purin-6-yl)amino­meth­yl]phen­yl}ethanone

**DOI:** 10.1107/S160053680900052X

**Published:** 2009-01-14

**Authors:** Michal Rouchal, Marek Nečas, Fabiana Pires de Carvalho, Robert Vícha

**Affiliations:** a Department of Chemistry, Faculty of Technology, Tomas Bata University in Zlin, Nám. T. G. Masaryka 275, Zlín,762 72, Czech Republic; b Department of Chemistry, Faculty of Science, Masaryk University in Brno, Kamenice 5, Brno-Bohunice, 625 00, Czech Republic

## Abstract

The structure of the title compound, C_27_H_32_ClN_5_O, consists of two crystallographically independent conformers differing slightly in all geometric parameters. Both contain nearly planar purine and benzene ring systems [maximum deviations of 0.046 (3) and 0.005 (2) Å, respectively], the dihedral angles between them being 76.44 (6) and 82.39 (6)°, and an adamantane cage consisting of three fused cyclo­hexane rings in almost ideal chair conformations, with C—C—C angles in the range 108.7 (2)–110.6 (2)°. The carbonyl plane and the benzene ring are almost coplanar [dihedral angles of 6.43 (9) and 0.64 (8)° in the two conformers]. The crystal structure is stabilized by inter­molecular N—H⋯N inter­actions that link adjacent mol­ecules into dimers and by some non-bonding contacts of the C—H⋯Cl type.

## Related literature

The title compound was prepared according to a modified procedure published by Fiorini & Abel (1989 ▶). For the synthesis and/or biological activity of related compounds, see: Veselý *et al.* (1994 ▶); Havlíček *et al.* (1997 ▶); de Azevedo *et al.* (1997 ▶); Kryštof *et al.* (2002 ▶); Kryštof *et al.* (2005 ▶); Legraverend & Grierson (2006 ▶). For some important properties of adamantane-bearing compounds, see: van Bommel *et al.* (2001 ▶); Cromwell *et al.* (1985 ▶). For related structures, see: Wang *et al.* (2001 ▶); Trávníček & Zatloukal (2004 ▶); Trávníček & Popa (2007*a* ▶,*b* ▶).
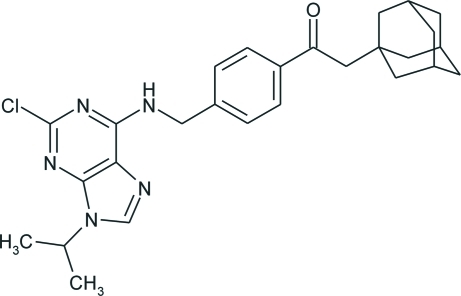


## Experimental

### 

#### Crystal data

C_27_H_32_ClN_5_O
*M* *_r_* = 478.03
Monoclinic, 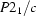

*a* = 15.8778 (5) Å
*b* = 20.2779 (5) Å
*c* = 15.2225 (5) Å
β = 104.233 (3)°
*V* = 4750.7 (3) Å^3^
*Z* = 8
Mo *K*α radiation
μ = 0.19 mm^−1^
*T* = 120 (2) K
0.50 × 0.40 × 0.30 mm

#### Data collection

Kuma KM-4 CCD diffractometer
Absorption correction: multi-scan (*Xcalibur*; Oxford Diffraction, 2006 ▶) *T*_min_ = 0.872, *T*_max_ = 0.944
47831 measured reflections
8353 independent reflections
5567 reflections with *I* > 2σ(*I*)
*R*_int_ = 0.026

#### Refinement

*R*[*F* ^2^ > 2σ(*F* ^2^)] = 0.042
*wR*(*F* ^2^) = 0.150
*S* = 1.09
8353 reflections
617 parameters
H-atom parameters constrained
Δρ_max_ = 0.35 e Å^−3^
Δρ_min_ = −0.29 e Å^−3^

### 

Data collection: *Xcalibur* (Oxford Diffraction, 2006 ▶); cell refinement: *Xcalibur*; data reduction: *Xcalibur*; program(s) used to solve structure: *SHELXS97* (Sheldrick, 2008 ▶); program(s) used to refine structure: *SHELXL97* (Sheldrick, 2008 ▶); molecular graphics: *ORTEP-3* (Farrugia, 1997 ▶); software used to prepare material for publication: *SHELXL97*.

## Supplementary Material

Crystal structure: contains datablocks global, I. DOI: 10.1107/S160053680900052X/pk2141sup1.cif

Structure factors: contains datablocks I. DOI: 10.1107/S160053680900052X/pk2141Isup2.hkl

Additional supplementary materials:  crystallographic information; 3D view; checkCIF report

## Figures and Tables

**Table 1:** Hydrogen-bond geometry (Å, °)

*D*—H⋯*A*	*D*—H	H⋯*A*	*D*⋯*A*	*D*—H⋯*A*
N5—H5*A*⋯N53^i^	0.88	2.20	2.997 (3)	150
N55—H55*A*⋯N3^ii^	0.88	2.18	2.946 (3)	145
C27—H27*A*⋯Cl1^iii^	0.98	2.86	3.732 (3)	149
C54—H54*B*⋯Cl51^iv^	0.99	2.76	3.698 (3)	158

Symmetry codes: (i) 
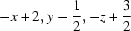
; (ii) 
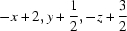
; (iii) 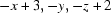; (iv) 
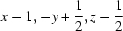
.

**Table 2:** Comparative torsion angles (°) for selected 2,6,9-trisubstituted purines containing the 2-chloro 6-benzylamino and 9-isopropyl unit

Compound	angle	value	angle	value
NG38^*a*^	C6—N6—C9—C10	115.22 (13)	H17—C17—N9—C4	−13.38 (18)
CIBAP1^*b*^	C6—N6—C9—C10	178.97 (15)	H16—C16—N9—C4	−63.03 (2)
CIBAP2^*c*^	C6—N6—C9—C10	−117.35 (2)	H16—C16—N9—C4	30.35 (3)
CIABAP^*d*^	C20—N5—C19—C16	100.28 (3)	H25—C25—N4—C22	45.01 (3)
CIABAP^*d*^	C70—N55—C69—C66	−99.62 (3)	H75—C75—N54—C72	−40.79 (3)

Notes: (*a*) Trávníček & Zatloukal (2004 ▶), where NG38 is *N*-[(2-azepan-1-yl)-9-isopropyl-9*H*-purin-6-yl]-4-methoxybenzylamine; (*b*) Trávníček & Popa (2007*a* ▶), where CIBAP1 is 2-chloro-6-[(2,6-dimethoxybenzyl)amino]-9-isopropylpurine; (*c*) Trávníček & Popa (2007*b* ▶), where CIBAP2 is 2-chloro-6-[(4-hydroxy-3,5-dimethoxybenzyl)amino]-9-isopropylpurine; (*d*) this work, where CIABAP is the title compound (the structure consists of two crystallographically independent molecules).

**Fig. 1.**
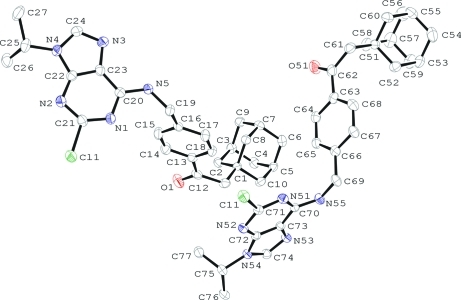
ORTEP of the asymmetric unit with atoms represented as 50% probability ellipsoids. H-atoms have been omitted to enhance clarity.

**Fig. 2.**
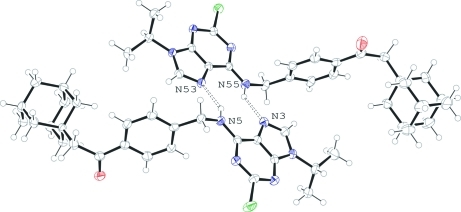
Part of the crystal structure of the title compound, showing the hydrogen bonding (dashed lines).

## supplementary crystallographic information

### Comment

The title molecule is related to the family of 2,6,9-trisubstituted purines that behave as potent inhibitors of cyclin-dependent kinases and show anticancer activity. The antiproliferative and proapoptotic effects of these drugs have been studied extensively with both an important and a promising result (Veselý *et al.*, 1994; Havlíček *et al.*, 1997; de Azevedo *et al.*, 1997; Kryštof *et al.*, 2002; Kryštof *et al.*, 2005; Legraverend & Grierson, 2006). The adamantane group is frequently used to improve the pharmacological properties of potential drugs. Whereas the lipophilic adamantane cage itself may increase solubility in non-polar systems (*e.g.* cell membranes), the non-covalent complex of adamantane with cyclodextrins can enhance solubility in water based media (Cromwell *et al.*, 1985; van Bommel *et al.*, 2001). Both these facilities have considerable importance in drug design and formulation. To the best of our knowledge, the title compound is the first described derivative of a 2,6,9-trisubstituted purine with an adamantyl group linked to 6-benzylamino substituent.

The structure consists of two crystallographically independent molecules slightly variant in geometry. Each ring is essentially planar, the maximum deviations from the best planes being 0.026 (2) Å for atom C23 (pyrimidine rings), 0.0050 (10) Å for atoms C72 and C73 (imidazole rings) and 0.005 (2) Å for atoms C13, C16 and C68 (benzene rings). The dihedral angles between purine and benzene rings are 76.44 (6)° and 82.39 (6)° respectively. The torsion angles C19–N5–C20–C23, C20–N5–C19–C16, N5–C19–C16–C17, C18–C13–C12–C11 and C13–C12–C11–C1 are 172.35 (2), 100.28 (3), 146.27 (2), -6.92 (4) and -100.18 (3)° respectively. The corresponding values of torsion angles for the second distinct conformer are -176.98 (2), -99.62 (3), -168.68 (2), 0.16 (4) and 95.57 (3) respectively. Comparative torsion angles for selected related molecules are shown in Table 2. The crystal structure is stabilized by intermolecular N–H···N interactions that link the molecules into pairs (Fig. 2 and Table 1), the N···N distances being 2.997 (3) and 2.946 (3) Å. respectively. There are also some additional intermolecular non-bonding contacts of the type C–H···Cl (Table 1). One from the two conformers is linked by C27–H27A···Cl1 interaction into pairs and the second conformer is linked by C54–H54B···Cl51 into linear chains. No other short intermolecular interactions were found.

### Experimental

The title compound was prepared according to a slightly modified literature procedure (Fiorini & Abel, 1989). 2,6-Dichloro-9-(propan-2-yl)-9*H*-purine (0.65 mmol, 150 mg) and 1-[4-(aminoethyl)phenyl)]-2-(1-adamantyl)ethanone hydrochloride (0.68 mmol, 218 mg) were dissolved in the mixture of DMF (2 cm^3^) and triethylamine (1.30 mmol, 0.18 cm^3^). The resulting solution was stirred and refluxed for 2.5 h. After the starting material had all reacted (according to TLC), the mixture was diluted with water and extracted five times with 15 cm^3^ of diethyl ether. The combined organic layers were washed twice with brine, dried over sodium sulfate and evaporated in vacuum. The crude product was purified by column chromatography (silica gel; petroleum ether/ethyl acetate, *v*/*v*, 1/1). The desired product was obtained as pale yellow crystalline powder (228 mg, 74%, mp 146–150°C). The crystal used for data collection was grown by liquid diffusion (acetone/hexane, *v*/*v*, 1/3) at -18°C within 48 h.

### Crystal data

**Table:**

C_27_H_32_ClN_5_O	*F*(000) = 2032
*M**_r_* = 478.03	*D*_x_ = 1.337 Mg m^−^^3^
Monoclinic, *P*2_1_/*c*	Melting point: 148 K
Hall symbol: -P 2ybc	Mo *K*α radiation, λ = 0.71073 Å
*a* = 15.8778 (5) Å	Cell parameters from 8353 reflections
*b* = 20.2779 (5) Å	θ = 2.8–25.0°
*c* = 15.2225 (5) Å	µ = 0.19 mm^−^^1^
β = 104.233 (3)°	*T* = 120 K
*V* = 4750.7 (3) Å^3^	Block, yellow
*Z* = 8	0.50 × 0.40 × 0.30 mm

### Data collection

**Table:**

Kuma KM-4 CCD diffractometer	8353 independent reflections
Radiation source: fine-focus sealed tube	5567 reflections with *I* > 2σ(*I*)
graphite	*R*_int_ = 0.026
Detector resolution: 0.06 pixels mm^-1^	θ_max_ = 25.0°, θ_min_ = 2.8°
ω scans	*h* = −16→18
Absorption correction: multi-scan (Xcalibur; Oxford Diffraction, 2006)	*k* = −24→23
*T*_min_ = 0.872, *T*_max_ = 0.944	*l* = −18→18
47831 measured reflections	

### Refinement

**Table:**

Refinement on *F*^2^	Primary atom site location: structure-invariant direct methods
Least-squares matrix: full	Secondary atom site location: difference Fourier map
*R*[*F*^2^ > 2σ(*F*^2^)] = 0.042	Hydrogen site location: inferred from neighbouring sites
*wR*(*F*^2^) = 0.150	H-atom parameters constrained
*S* = 1.09	*w* = 1/[σ^2^(*F*_o_^2^) + (0.0761*P*)^2^ + 2.6597*P*] where *P* = (*F*_o_^2^ + 2*F*_c_^2^)/3
8353 reflections	(Δ/σ)_max_ = 0.005
617 parameters	Δρ_max_ = 0.35 e Å^−^^3^
0 restraints	Δρ_min_ = −0.29 e Å^−^^3^

### Special details

**Table:**

Geometry. All e.s.d.'s (except the e.s.d. in the dihedral angle between two l.s. planes) are estimated using the full covariance matrix. The cell e.s.d.'s are taken into account individually in the estimation of e.s.d.'s in distances, angles and torsion angles; correlations between e.s.d.'s in cell parameters are only used when they are defined by crystal symmetry. An approximate (isotropic) treatment of cell e.s.d.'s is used for estimating e.s.d.'s involving l.s. planes.
Refinement. Refinement of *F*^2^ against ALL reflections. The weighted *R*-factor *wR* and goodness of fit *S* are based on *F*^2^, conventional *R*-factors *R* are based on *F*, with *F* set to zero for negative *F*^2^. The threshold expression of *F*^2^ > 2σ(*F*^2^) is used only for calculating *R*-factors(gt) *etc*. and is not relevant to the choice of reflections for refinement. *R*-factors based on *F*^2^ are statistically about twice as large as those based on *F*, and *R*- factors based on ALL data will be even larger.

### Fractional atomic coordinates and isotropic or equivalent isotropic displacement parameters (Å^2^)

**Table:**

	*x*	*y*	*z*	*U*_iso_*/*U*_eq_	
Cl1	1.54371 (4)	0.16850 (4)	1.01111 (5)	0.0416 (2)	
O1	1.15991 (12)	0.38506 (9)	0.65047 (14)	0.0418 (5)	
N1	1.39808 (13)	0.11570 (10)	0.92710 (14)	0.0265 (5)	
N2	1.53027 (13)	0.05913 (10)	0.92127 (14)	0.0278 (5)	
N4	1.50180 (14)	−0.04121 (10)	0.83228 (14)	0.0289 (5)	
N3	1.35644 (14)	−0.03462 (10)	0.78459 (15)	0.0306 (5)	
N5	1.26108 (13)	0.07666 (10)	0.85571 (14)	0.0261 (5)	
H5A	1.2293	0.0495	0.8159	0.031*	
C1	0.95337 (16)	0.38700 (11)	0.59602 (15)	0.0222 (5)	
C2	0.98831 (16)	0.39531 (12)	0.51181 (17)	0.0269 (6)	
H2B	1.0340	0.3620	0.5123	0.032*	
H2C	1.0147	0.4396	0.5122	0.032*	
C3	0.91501 (17)	0.38722 (12)	0.42610 (17)	0.0281 (6)	
H3A	0.9391	0.3929	0.3717	0.034*	
C4	0.84508 (17)	0.43886 (13)	0.42383 (18)	0.0321 (6)	
H4B	0.7976	0.4333	0.3684	0.038*	
H4C	0.8699	0.4836	0.4228	0.038*	
C5	0.80936 (17)	0.43108 (13)	0.50779 (17)	0.0285 (6)	
H5B	0.7636	0.4651	0.5070	0.034*	
C6	0.77037 (17)	0.36252 (13)	0.50670 (17)	0.0302 (6)	
H6A	0.7225	0.3571	0.4516	0.036*	
H6B	0.7461	0.3569	0.5602	0.036*	
C7	0.83958 (17)	0.31037 (13)	0.50795 (17)	0.0282 (6)	
H7A	0.8132	0.2656	0.5074	0.034*	
C8	0.91320 (16)	0.31812 (12)	0.59364 (16)	0.0254 (6)	
H8A	0.9583	0.2842	0.5944	0.030*	
H8B	0.8902	0.3117	0.6479	0.030*	
C9	0.87618 (17)	0.31819 (13)	0.42455 (17)	0.0308 (6)	
H9A	0.9215	0.2845	0.4253	0.037*	
H9B	0.8294	0.3121	0.3687	0.037*	
C10	0.88214 (17)	0.43880 (12)	0.59306 (17)	0.0284 (6)	
H10A	0.8584	0.4338	0.6470	0.034*	
H10B	0.9075	0.4835	0.5946	0.034*	
C11	1.02429 (17)	0.39841 (12)	0.68438 (17)	0.0291 (6)	
H11A	1.0369	0.4462	0.6908	0.035*	
H11B	1.0011	0.3847	0.7363	0.035*	
C12	1.10835 (17)	0.36200 (13)	0.68945 (17)	0.0287 (6)	
C13	1.12982 (16)	0.29932 (12)	0.74137 (16)	0.0258 (6)	
C14	1.20554 (18)	0.26697 (13)	0.73665 (18)	0.0331 (6)	
H14A	1.2396	0.2843	0.6987	0.040*	
C15	1.23249 (17)	0.21078 (13)	0.78510 (18)	0.0327 (6)	
H15A	1.2847	0.1898	0.7804	0.039*	
C16	1.18432 (16)	0.18431 (12)	0.84080 (16)	0.0240 (5)	
C17	1.10862 (16)	0.21535 (13)	0.84536 (17)	0.0279 (6)	
H17A	1.0745	0.1974	0.8828	0.033*	
C18	1.08111 (16)	0.27234 (12)	0.79635 (17)	0.0280 (6)	
H18A	1.0285	0.2930	0.8005	0.034*	
C19	1.21638 (16)	0.12463 (12)	0.89844 (17)	0.0263 (6)	
H19A	1.2563	0.1394	0.9557	0.032*	
H19B	1.1662	0.1029	0.9140	0.032*	
C20	1.34751 (16)	0.07103 (12)	0.87280 (15)	0.0228 (5)	
C21	1.48320 (16)	0.10626 (13)	0.94474 (16)	0.0275 (6)	
C22	1.47769 (16)	0.01555 (12)	0.86771 (16)	0.0246 (6)	
C23	1.38849 (16)	0.01919 (12)	0.83828 (17)	0.0255 (6)	
C24	1.42650 (18)	−0.06881 (13)	0.78385 (19)	0.0333 (6)	
H24A	1.4248	−0.1094	0.7522	0.040*	
C25	1.59119 (17)	−0.06731 (13)	0.84933 (18)	0.0326 (6)	
H25A	1.6181	−0.0638	0.9158	0.039*	
C26	1.64435 (18)	−0.02650 (14)	0.8010 (2)	0.0389 (7)	
H26A	1.6197	−0.0295	0.7355	0.058*	
H26B	1.7043	−0.0428	0.8157	0.058*	
H26C	1.6438	0.0196	0.8202	0.058*	
C27	1.5911 (2)	−0.13913 (15)	0.8235 (3)	0.0566 (10)	
H27A	1.5524	−0.1637	0.8528	0.085*	
H27B	1.6502	−0.1568	0.8433	0.085*	
H27C	1.5706	−0.1435	0.7575	0.085*	
Cl51	1.06711 (4)	0.32556 (3)	1.02370 (4)	0.03138 (18)	
O51	0.65672 (14)	0.16100 (11)	0.55786 (14)	0.0508 (6)	
N51	0.92396 (13)	0.37756 (10)	0.93336 (13)	0.0251 (5)	
N52	1.05595 (13)	0.43726 (10)	0.93901 (13)	0.0231 (5)	
N53	0.88368 (13)	0.53117 (10)	0.79834 (13)	0.0237 (5)	
N54	1.02812 (13)	0.53960 (10)	0.85313 (13)	0.0223 (5)	
N55	0.78821 (13)	0.41714 (10)	0.86172 (13)	0.0264 (5)	
H55A	0.7566	0.4474	0.8274	0.032*	
C51	0.47184 (16)	0.11567 (12)	0.58865 (16)	0.0259 (6)	
C52	0.43209 (18)	0.16706 (13)	0.63953 (18)	0.0326 (6)	
H52B	0.4403	0.1533	0.7035	0.039*	
H52C	0.4618	0.2099	0.6388	0.039*	
C53	0.33568 (18)	0.17478 (14)	0.5956 (2)	0.0370 (7)	
H53B	0.3103	0.2083	0.6298	0.044*	
C54	0.2906 (2)	0.10865 (16)	0.5982 (2)	0.0478 (8)	
H54A	0.2991	0.0938	0.6617	0.057*	
H54B	0.2274	0.1133	0.5712	0.057*	
C55	0.3289 (2)	0.05839 (15)	0.5449 (2)	0.0496 (8)	
H55B	0.2988	0.0151	0.5453	0.060*	
C56	0.3168 (2)	0.08135 (18)	0.4476 (2)	0.0605 (10)	
H56A	0.3407	0.0479	0.4129	0.073*	
H56B	0.2541	0.0867	0.4188	0.073*	
C57	0.3632 (2)	0.14650 (17)	0.44614 (19)	0.0457 (8)	
H57A	0.3556	0.1613	0.3820	0.055*	
C58	0.45957 (18)	0.13811 (15)	0.49055 (18)	0.0373 (7)	
H58A	0.4901	0.1805	0.4890	0.045*	
H58B	0.4849	0.1050	0.4567	0.045*	
C59	0.3243 (2)	0.19791 (16)	0.49812 (19)	0.0437 (8)	
H59A	0.3539	0.2408	0.4973	0.052*	
H59B	0.2618	0.2039	0.4690	0.052*	
C60	0.42482 (18)	0.05001 (13)	0.5893 (2)	0.0380 (7)	
H60A	0.4500	0.0164	0.5560	0.046*	
H60B	0.4324	0.0347	0.6525	0.046*	
C61	0.56868 (17)	0.10361 (13)	0.63550 (19)	0.0329 (6)	
H61A	0.5739	0.0958	0.7008	0.039*	
H61B	0.5877	0.0629	0.6101	0.039*	
C62	0.62922 (17)	0.15816 (14)	0.62633 (19)	0.0344 (7)	
C63	0.65636 (16)	0.20984 (13)	0.69797 (17)	0.0279 (6)	
C68	0.62858 (17)	0.21168 (13)	0.77775 (17)	0.0311 (6)	
H68A	0.5905	0.1786	0.7895	0.037*	
C67	0.65643 (17)	0.26183 (13)	0.84013 (17)	0.0294 (6)	
H67A	0.6364	0.2632	0.8940	0.035*	
C66	0.71300 (16)	0.30974 (12)	0.82460 (16)	0.0247 (6)	
C65	0.74072 (16)	0.30712 (13)	0.74527 (17)	0.0281 (6)	
H65A	0.7795	0.3399	0.7339	0.034*	
C64	0.71310 (17)	0.25799 (13)	0.68273 (17)	0.0295 (6)	
H64A	0.7330	0.2570	0.6288	0.035*	
C69	0.74333 (17)	0.36378 (13)	0.89382 (16)	0.0276 (6)	
H69A	0.6923	0.3819	0.9120	0.033*	
H69B	0.7826	0.3443	0.9484	0.033*	
C70	0.87432 (15)	0.42347 (11)	0.88074 (15)	0.0218 (5)	
C71	1.00903 (16)	0.38826 (12)	0.95686 (15)	0.0230 (5)	
C72	1.00400 (16)	0.48160 (11)	0.88524 (15)	0.0216 (5)	
C73	0.91542 (16)	0.47738 (11)	0.85193 (15)	0.0214 (5)	
C74	0.95342 (16)	0.56661 (12)	0.80151 (16)	0.0259 (6)	
H74A	0.9521	0.6075	0.7706	0.031*	
C75	1.11732 (16)	0.56615 (12)	0.87100 (16)	0.0250 (6)	
H75A	1.1462	0.5577	0.9362	0.030*	
C76	1.11638 (17)	0.63973 (12)	0.85592 (18)	0.0311 (6)	
H76A	1.0919	0.6492	0.7916	0.047*	
H76B	1.1759	0.6568	0.8743	0.047*	
H76C	1.0808	0.6609	0.8921	0.047*	
C77	1.16747 (17)	0.52945 (13)	0.81434 (18)	0.0308 (6)	
H77A	1.1671	0.4822	0.8277	0.046*	
H77B	1.2275	0.5455	0.8285	0.046*	
H77C	1.1404	0.5368	0.7500	0.046*	

### Atomic displacement parameters (Å^2^)

**Table:**

	*U*^11^	*U*^22^	*U*^33^	*U*^12^	*U*^13^	*U*^23^
Cl1	0.0251 (4)	0.0477 (4)	0.0511 (4)	−0.0055 (3)	0.0075 (3)	−0.0211 (3)
O1	0.0265 (11)	0.0396 (12)	0.0568 (13)	−0.0032 (9)	0.0051 (10)	0.0171 (10)
N1	0.0216 (12)	0.0287 (12)	0.0294 (11)	0.0004 (9)	0.0067 (9)	0.0004 (9)
N2	0.0225 (12)	0.0299 (12)	0.0305 (11)	0.0001 (9)	0.0057 (9)	0.0000 (9)
N4	0.0235 (12)	0.0237 (11)	0.0394 (12)	0.0046 (9)	0.0079 (10)	0.0022 (9)
N3	0.0285 (13)	0.0233 (12)	0.0387 (12)	0.0021 (9)	0.0055 (10)	0.0002 (10)
N5	0.0201 (12)	0.0246 (11)	0.0319 (11)	0.0002 (9)	0.0030 (9)	−0.0023 (9)
C1	0.0228 (13)	0.0199 (12)	0.0232 (12)	0.0009 (10)	0.0045 (10)	−0.0027 (10)
C2	0.0238 (14)	0.0245 (14)	0.0326 (14)	−0.0020 (11)	0.0074 (11)	0.0031 (11)
C3	0.0293 (15)	0.0328 (15)	0.0232 (12)	−0.0005 (11)	0.0081 (11)	0.0013 (11)
C4	0.0291 (15)	0.0322 (15)	0.0337 (14)	−0.0007 (12)	0.0055 (12)	0.0043 (12)
C5	0.0252 (14)	0.0265 (14)	0.0324 (14)	0.0075 (11)	0.0043 (11)	−0.0013 (11)
C6	0.0226 (14)	0.0416 (16)	0.0271 (13)	−0.0001 (12)	0.0072 (11)	0.0015 (12)
C7	0.0285 (15)	0.0241 (13)	0.0314 (14)	−0.0054 (11)	0.0061 (11)	−0.0007 (11)
C8	0.0265 (14)	0.0230 (13)	0.0277 (13)	0.0006 (11)	0.0088 (11)	0.0028 (10)
C9	0.0271 (15)	0.0347 (15)	0.0276 (13)	0.0001 (12)	0.0010 (11)	−0.0084 (11)
C10	0.0310 (15)	0.0253 (14)	0.0281 (13)	0.0049 (11)	0.0060 (11)	−0.0057 (11)
C11	0.0306 (15)	0.0243 (13)	0.0285 (13)	0.0017 (11)	0.0000 (11)	−0.0031 (11)
C12	0.0242 (14)	0.0283 (14)	0.0297 (13)	−0.0047 (11)	−0.0007 (11)	−0.0025 (11)
C13	0.0252 (14)	0.0241 (13)	0.0263 (13)	−0.0019 (11)	0.0027 (11)	−0.0048 (10)
C14	0.0313 (16)	0.0327 (15)	0.0393 (15)	−0.0010 (12)	0.0165 (12)	0.0056 (12)
C15	0.0236 (14)	0.0309 (15)	0.0462 (16)	0.0043 (11)	0.0137 (12)	0.0078 (12)
C16	0.0202 (13)	0.0271 (13)	0.0237 (12)	−0.0045 (10)	0.0034 (10)	−0.0063 (10)
C17	0.0248 (14)	0.0332 (15)	0.0286 (13)	0.0004 (11)	0.0122 (11)	0.0025 (11)
C18	0.0206 (13)	0.0294 (14)	0.0336 (14)	0.0041 (11)	0.0061 (11)	−0.0032 (11)
C19	0.0218 (14)	0.0282 (14)	0.0296 (13)	0.0033 (11)	0.0074 (11)	0.0003 (11)
C20	0.0247 (14)	0.0213 (13)	0.0220 (12)	0.0000 (10)	0.0051 (10)	0.0034 (10)
C21	0.0226 (14)	0.0333 (15)	0.0256 (13)	−0.0025 (11)	0.0039 (11)	−0.0014 (11)
C22	0.0268 (15)	0.0218 (13)	0.0254 (12)	0.0033 (11)	0.0066 (11)	0.0032 (10)
C23	0.0219 (14)	0.0245 (13)	0.0307 (13)	0.0008 (10)	0.0073 (11)	0.0058 (11)
C24	0.0314 (16)	0.0229 (14)	0.0441 (16)	0.0010 (12)	0.0063 (13)	−0.0036 (12)
C25	0.0267 (15)	0.0363 (15)	0.0357 (14)	0.0108 (12)	0.0097 (12)	0.0078 (12)
C26	0.0286 (16)	0.0364 (16)	0.0519 (18)	0.0054 (12)	0.0101 (13)	0.0125 (13)
C27	0.047 (2)	0.0323 (17)	0.104 (3)	0.0129 (15)	0.044 (2)	0.0069 (17)
Cl51	0.0289 (4)	0.0282 (4)	0.0354 (4)	0.0007 (3)	0.0048 (3)	0.0088 (3)
O51	0.0413 (13)	0.0690 (15)	0.0482 (13)	−0.0183 (11)	0.0224 (11)	−0.0241 (11)
N51	0.0265 (12)	0.0255 (11)	0.0229 (10)	−0.0025 (9)	0.0055 (9)	0.0000 (9)
N52	0.0223 (11)	0.0235 (11)	0.0227 (10)	−0.0015 (9)	0.0042 (9)	−0.0004 (8)
N53	0.0227 (12)	0.0232 (11)	0.0243 (10)	−0.0024 (9)	0.0041 (9)	0.0010 (8)
N54	0.0200 (11)	0.0224 (11)	0.0240 (10)	−0.0027 (8)	0.0044 (9)	0.0007 (8)
N55	0.0247 (12)	0.0270 (12)	0.0273 (11)	−0.0024 (9)	0.0060 (9)	0.0043 (9)
C51	0.0247 (14)	0.0229 (13)	0.0295 (13)	−0.0003 (10)	0.0053 (11)	−0.0010 (10)
C52	0.0335 (16)	0.0294 (15)	0.0335 (14)	0.0035 (12)	0.0057 (12)	−0.0011 (11)
C53	0.0293 (16)	0.0377 (16)	0.0448 (16)	0.0082 (12)	0.0106 (13)	−0.0017 (13)
C54	0.0282 (17)	0.056 (2)	0.061 (2)	0.0011 (14)	0.0141 (15)	0.0089 (16)
C55	0.0321 (18)	0.0376 (18)	0.079 (2)	−0.0115 (14)	0.0124 (16)	−0.0095 (16)
C56	0.039 (2)	0.074 (3)	0.057 (2)	0.0038 (17)	−0.0099 (16)	−0.0293 (18)
C57	0.0411 (18)	0.066 (2)	0.0263 (14)	0.0110 (16)	0.0002 (13)	0.0028 (14)
C58	0.0352 (17)	0.0457 (18)	0.0331 (15)	0.0060 (13)	0.0123 (13)	0.0005 (13)
C59	0.0400 (18)	0.0469 (18)	0.0419 (17)	0.0134 (14)	0.0056 (14)	0.0094 (14)
C60	0.0375 (17)	0.0240 (14)	0.0537 (18)	−0.0033 (12)	0.0132 (14)	−0.0003 (13)
C61	0.0313 (16)	0.0275 (14)	0.0383 (15)	0.0053 (12)	0.0054 (12)	−0.0024 (12)
C62	0.0217 (15)	0.0425 (17)	0.0372 (16)	0.0028 (12)	0.0038 (12)	−0.0049 (12)
C63	0.0238 (14)	0.0313 (14)	0.0272 (13)	0.0070 (11)	0.0037 (11)	0.0027 (11)
C68	0.0257 (14)	0.0309 (15)	0.0338 (14)	−0.0035 (11)	0.0018 (11)	0.0078 (12)
C67	0.0257 (14)	0.0353 (15)	0.0266 (13)	−0.0043 (11)	0.0051 (11)	0.0058 (11)
C66	0.0206 (13)	0.0250 (13)	0.0253 (12)	0.0034 (10)	−0.0008 (10)	0.0060 (10)
C65	0.0245 (14)	0.0282 (14)	0.0336 (14)	0.0004 (11)	0.0108 (11)	0.0031 (11)
C64	0.0280 (15)	0.0327 (15)	0.0302 (14)	0.0014 (11)	0.0116 (12)	0.0020 (11)
C69	0.0245 (14)	0.0345 (15)	0.0238 (12)	−0.0065 (11)	0.0058 (11)	0.0051 (11)
C70	0.0215 (14)	0.0234 (13)	0.0212 (12)	−0.0024 (10)	0.0066 (10)	−0.0054 (10)
C71	0.0253 (14)	0.0236 (13)	0.0200 (12)	−0.0011 (10)	0.0051 (10)	−0.0001 (10)
C72	0.0254 (14)	0.0221 (13)	0.0184 (11)	−0.0007 (10)	0.0073 (10)	−0.0032 (10)
C73	0.0242 (14)	0.0222 (13)	0.0188 (11)	−0.0017 (10)	0.0069 (10)	−0.0023 (10)
C74	0.0261 (14)	0.0246 (13)	0.0266 (13)	−0.0002 (11)	0.0056 (11)	0.0002 (10)
C75	0.0211 (14)	0.0286 (14)	0.0245 (12)	−0.0043 (10)	0.0038 (10)	0.0029 (10)
C76	0.0277 (15)	0.0275 (14)	0.0384 (15)	−0.0088 (11)	0.0087 (12)	−0.0009 (12)
C77	0.0246 (15)	0.0313 (15)	0.0363 (15)	−0.0017 (11)	0.0069 (12)	0.0018 (11)

### Geometric parameters (Å, °)

**Table:**

Cl1—C21	1.749 (3)	Cl51—C71	1.744 (2)
O1—C12	1.217 (3)	O51—C62	1.226 (3)
N1—C21	1.326 (3)	N51—C71	1.327 (3)
N1—C20	1.350 (3)	N51—C70	1.349 (3)
N2—C21	1.316 (3)	N52—C71	1.310 (3)
N2—C22	1.345 (3)	N52—C72	1.351 (3)
N4—C24	1.361 (3)	N53—C74	1.311 (3)
N4—C22	1.365 (3)	N53—C73	1.381 (3)
N4—C25	1.477 (3)	N54—C72	1.364 (3)
N3—C24	1.313 (3)	N54—C74	1.366 (3)
N3—C23	1.383 (3)	N54—C75	1.477 (3)
N5—C20	1.337 (3)	N55—C70	1.332 (3)
N5—C19	1.449 (3)	N55—C69	1.445 (3)
N5—H5A	0.8800	N55—H55A	0.8800
C1—C2	1.526 (3)	C51—C52	1.525 (4)
C1—C8	1.532 (3)	C51—C58	1.527 (4)
C1—C10	1.536 (3)	C51—C60	1.528 (4)
C1—C11	1.545 (3)	C51—C61	1.547 (4)
C2—C3	1.529 (3)	C52—C53	1.521 (4)
C2—H2B	0.9900	C52—H52B	0.9900
C2—H2C	0.9900	C52—H52C	0.9900
C3—C4	1.520 (4)	C53—C59	1.524 (4)
C3—C9	1.527 (4)	C53—C54	1.525 (4)
C3—H3A	1.0000	C53—H53B	1.0000
C4—C5	1.528 (4)	C54—C55	1.520 (4)
C4—H4B	0.9900	C54—H54A	0.9900
C4—H4C	0.9900	C54—H54B	0.9900
C5—C10	1.519 (3)	C55—C60	1.516 (4)
C5—C6	1.520 (4)	C55—C56	1.518 (5)
C5—H5B	1.0000	C55—H55B	1.0000
C6—C7	1.522 (4)	C56—C57	1.515 (5)
C6—H6A	0.9900	C56—H56A	0.9900
C6—H6B	0.9900	C56—H56B	0.9900
C7—C9	1.529 (4)	C57—C58	1.523 (4)
C7—C8	1.531 (3)	C57—C59	1.528 (4)
C7—H7A	1.0000	C57—H57A	1.0000
C8—H8A	0.9900	C58—H58A	0.9900
C8—H8B	0.9900	C58—H58B	0.9900
C9—H9A	0.9900	C59—H59A	0.9900
C9—H9B	0.9900	C59—H59B	0.9900
C10—H10A	0.9900	C60—H60A	0.9900
C10—H10B	0.9900	C60—H60B	0.9900
C11—C12	1.511 (4)	C61—C62	1.494 (4)
C11—H11A	0.9900	C61—H61A	0.9900
C11—H11B	0.9900	C61—H61B	0.9900
C12—C13	1.491 (4)	C62—C63	1.497 (4)
C13—C18	1.384 (4)	C63—C64	1.386 (4)
C13—C14	1.387 (4)	C63—C68	1.391 (4)
C14—C15	1.368 (4)	C68—C67	1.387 (4)
C14—H14A	0.9500	C68—H68A	0.9500
C15—C16	1.382 (4)	C67—C66	1.383 (3)
C15—H15A	0.9500	C67—H67A	0.9500
C16—C17	1.374 (3)	C66—C65	1.384 (4)
C16—C19	1.508 (3)	C66—C69	1.514 (3)
C17—C18	1.386 (4)	C65—C64	1.373 (4)
C17—H17A	0.9500	C65—H65A	0.9500
C18—H18A	0.9500	C64—H64A	0.9500
C19—H19A	0.9900	C69—H69A	0.9900
C19—H19B	0.9900	C69—H69B	0.9900
C20—C23	1.405 (3)	C70—C73	1.398 (3)
C22—C23	1.378 (4)	C72—C73	1.375 (3)
C24—H24A	0.9500	C74—H74A	0.9500
C25—C26	1.498 (4)	C75—C77	1.507 (4)
C25—C27	1.508 (4)	C75—C76	1.509 (3)
C25—H25A	1.0000	C75—H75A	1.0000
C26—H26A	0.9800	C76—H76A	0.9800
C26—H26B	0.9800	C76—H76B	0.9800
C26—H26C	0.9800	C76—H76C	0.9800
C27—H27A	0.9800	C77—H77A	0.9800
C27—H27B	0.9800	C77—H77B	0.9800
C27—H27C	0.9800	C77—H77C	0.9800
			
C21—N1—C20	116.8 (2)	C71—N51—C70	117.0 (2)
C21—N2—C22	109.4 (2)	C71—N52—C72	109.7 (2)
C24—N4—C22	105.5 (2)	C74—N53—C73	103.2 (2)
C24—N4—C25	128.8 (2)	C72—N54—C74	105.7 (2)
C22—N4—C25	125.7 (2)	C72—N54—C75	126.1 (2)
C24—N3—C23	103.4 (2)	C74—N54—C75	128.1 (2)
C20—N5—C19	124.2 (2)	C70—N55—C69	124.3 (2)
C20—N5—H5A	117.9	C70—N55—H55A	117.8
C19—N5—H5A	117.9	C69—N55—H55A	117.8
C2—C1—C8	108.42 (19)	C52—C51—C58	108.9 (2)
C2—C1—C10	108.5 (2)	C52—C51—C60	109.2 (2)
C8—C1—C10	108.9 (2)	C58—C51—C60	108.7 (2)
C2—C1—C11	112.2 (2)	C52—C51—C61	111.3 (2)
C8—C1—C11	111.55 (19)	C58—C51—C61	111.8 (2)
C10—C1—C11	107.27 (19)	C60—C51—C61	106.9 (2)
C1—C2—C3	110.3 (2)	C53—C52—C51	110.2 (2)
C1—C2—H2B	109.6	C53—C52—H52B	109.6
C3—C2—H2B	109.6	C51—C52—H52B	109.6
C1—C2—H2C	109.6	C53—C52—H52C	109.6
C3—C2—H2C	109.6	C51—C52—H52C	109.6
H2B—C2—H2C	108.1	H52B—C52—H52C	108.1
C4—C3—C9	109.9 (2)	C52—C53—C59	109.0 (2)
C4—C3—C2	110.1 (2)	C52—C53—C54	109.1 (2)
C9—C3—C2	109.3 (2)	C59—C53—C54	110.6 (3)
C4—C3—H3A	109.1	C52—C53—H53B	109.4
C9—C3—H3A	109.1	C59—C53—H53B	109.4
C2—C3—H3A	109.1	C54—C53—H53B	109.4
C3—C4—C5	109.2 (2)	C55—C54—C53	108.9 (2)
C3—C4—H4B	109.8	C55—C54—H54A	109.9
C5—C4—H4B	109.8	C53—C54—H54A	109.9
C3—C4—H4C	109.8	C55—C54—H54B	109.9
C5—C4—H4C	109.8	C53—C54—H54B	109.9
H4B—C4—H4C	108.3	H54A—C54—H54B	108.3
C10—C5—C6	109.0 (2)	C60—C55—C56	110.0 (3)
C10—C5—C4	110.1 (2)	C60—C55—C54	109.1 (3)
C6—C5—C4	108.6 (2)	C56—C55—C54	110.1 (3)
C10—C5—H5B	109.7	C60—C55—H55B	109.2
C6—C5—H5B	109.7	C56—C55—H55B	109.2
C4—C5—H5B	109.7	C54—C55—H55B	109.2
C5—C6—C7	110.1 (2)	C57—C56—C55	109.7 (2)
C5—C6—H6A	109.6	C57—C56—H56A	109.7
C7—C6—H6A	109.6	C55—C56—H56A	109.7
C5—C6—H6B	109.6	C57—C56—H56B	109.7
C7—C6—H6B	109.6	C55—C56—H56B	109.7
H6A—C6—H6B	108.1	H56A—C56—H56B	108.2
C6—C7—C9	109.8 (2)	C56—C57—C58	109.6 (3)
C6—C7—C8	109.7 (2)	C56—C57—C59	109.1 (3)
C9—C7—C8	109.3 (2)	C58—C57—C59	109.6 (2)
C6—C7—H7A	109.4	C56—C57—H57A	109.5
C9—C7—H7A	109.4	C58—C57—H57A	109.5
C8—C7—H7A	109.4	C59—C57—H57A	109.5
C7—C8—C1	109.88 (19)	C57—C58—C51	109.9 (2)
C7—C8—H8A	109.7	C57—C58—H58A	109.7
C1—C8—H8A	109.7	C51—C58—H58A	109.7
C7—C8—H8B	109.7	C57—C58—H58B	109.7
C1—C8—H8B	109.7	C51—C58—H58B	109.7
H8A—C8—H8B	108.2	H58A—C58—H58B	108.2
C3—C9—C7	108.5 (2)	C53—C59—C57	109.1 (2)
C3—C9—H9A	110.0	C53—C59—H59A	109.9
C7—C9—H9A	110.0	C57—C59—H59A	109.9
C3—C9—H9B	110.0	C53—C59—H59B	109.9
C7—C9—H9B	110.0	C57—C59—H59B	109.9
H9A—C9—H9B	108.4	H59A—C59—H59B	108.3
C5—C10—C1	110.76 (19)	C55—C60—C51	109.8 (2)
C5—C10—H10A	109.5	C55—C60—H60A	109.7
C1—C10—H10A	109.5	C51—C60—H60A	109.7
C5—C10—H10B	109.5	C55—C60—H60B	109.7
C1—C10—H10B	109.5	C51—C60—H60B	109.7
H10A—C10—H10B	108.1	H60A—C60—H60B	108.2
C12—C11—C1	114.6 (2)	C62—C61—C51	115.4 (2)
C12—C11—H11A	108.6	C62—C61—H61A	108.4
C1—C11—H11A	108.6	C51—C61—H61A	108.4
C12—C11—H11B	108.6	C62—C61—H61B	108.4
C1—C11—H11B	108.6	C51—C61—H61B	108.4
H11A—C11—H11B	107.6	H61A—C61—H61B	107.5
O1—C12—C13	119.5 (2)	O51—C62—C61	119.1 (2)
O1—C12—C11	118.6 (2)	O51—C62—C63	119.2 (3)
C13—C12—C11	121.8 (2)	C61—C62—C63	121.8 (2)
C18—C13—C14	117.7 (2)	C64—C63—C68	119.1 (2)
C18—C13—C12	124.6 (2)	C64—C63—C62	117.0 (2)
C14—C13—C12	117.6 (2)	C68—C63—C62	124.0 (2)
C15—C14—C13	121.8 (2)	C67—C68—C63	120.1 (2)
C15—C14—H14A	119.1	C67—C68—H68A	120.0
C13—C14—H14A	119.1	C63—C68—H68A	120.0
C14—C15—C16	120.5 (2)	C66—C67—C68	120.6 (2)
C14—C15—H15A	119.8	C66—C67—H67A	119.7
C16—C15—H15A	119.8	C68—C67—H67A	119.7
C17—C16—C15	118.4 (2)	C67—C66—C65	118.8 (2)
C17—C16—C19	120.8 (2)	C67—C66—C69	119.9 (2)
C15—C16—C19	120.7 (2)	C65—C66—C69	121.3 (2)
C16—C17—C18	121.2 (2)	C64—C65—C66	121.1 (2)
C16—C17—H17A	119.4	C64—C65—H65A	119.4
C18—C17—H17A	119.4	C66—C65—H65A	119.4
C13—C18—C17	120.4 (2)	C65—C64—C63	120.3 (2)
C13—C18—H18A	119.8	C65—C64—H64A	119.8
C17—C18—H18A	119.8	C63—C64—H64A	119.8
N5—C19—C16	114.2 (2)	N55—C69—C66	113.9 (2)
N5—C19—H19A	108.7	N55—C69—H69A	108.8
C16—C19—H19A	108.7	C66—C69—H69A	108.8
N5—C19—H19B	108.7	N55—C69—H69B	108.8
C16—C19—H19B	108.7	C66—C69—H69B	108.8
H19A—C19—H19B	107.6	H69A—C69—H69B	107.7
N5—C20—N1	119.3 (2)	N55—C70—N51	118.8 (2)
N5—C20—C23	122.6 (2)	N55—C70—C73	122.7 (2)
N1—C20—C23	118.1 (2)	N51—C70—C73	118.4 (2)
N2—C21—N1	132.0 (2)	N52—C71—N51	131.4 (2)
N2—C21—Cl1	114.40 (19)	N52—C71—Cl51	115.34 (18)
N1—C21—Cl1	113.63 (19)	N51—C71—Cl51	113.28 (18)
N2—C22—N4	126.9 (2)	N52—C72—N54	127.4 (2)
N2—C22—C23	126.7 (2)	N52—C72—C73	126.8 (2)
N4—C22—C23	106.4 (2)	N54—C72—C73	105.9 (2)
C22—C23—N3	110.4 (2)	C72—C73—N53	111.1 (2)
C22—C23—C20	116.9 (2)	C72—C73—C70	116.5 (2)
N3—C23—C20	132.4 (2)	N53—C73—C70	132.3 (2)
N3—C24—N4	114.3 (2)	N53—C74—N54	114.0 (2)
N3—C24—H24A	122.9	N53—C74—H74A	123.0
N4—C24—H24A	122.9	N54—C74—H74A	123.0
N4—C25—C26	110.4 (2)	N54—C75—C77	109.4 (2)
N4—C25—C27	111.2 (2)	N54—C75—C76	111.1 (2)
C26—C25—C27	111.7 (2)	C77—C75—C76	112.8 (2)
N4—C25—H25A	107.8	N54—C75—H75A	107.8
C26—C25—H25A	107.8	C77—C75—H75A	107.8
C27—C25—H25A	107.8	C76—C75—H75A	107.8
C25—C26—H26A	109.5	C75—C76—H76A	109.5
C25—C26—H26B	109.5	C75—C76—H76B	109.5
H26A—C26—H26B	109.5	H76A—C76—H76B	109.5
C25—C26—H26C	109.5	C75—C76—H76C	109.5
H26A—C26—H26C	109.5	H76A—C76—H76C	109.5
H26B—C26—H26C	109.5	H76B—C76—H76C	109.5
C25—C27—H27A	109.5	C75—C77—H77A	109.5
C25—C27—H27B	109.5	C75—C77—H77B	109.5
H27A—C27—H27B	109.5	H77A—C77—H77B	109.5
C25—C27—H27C	109.5	C75—C77—H77C	109.5
H27A—C27—H27C	109.5	H77A—C77—H77C	109.5
H27B—C27—H27C	109.5	H77B—C77—H77C	109.5
			
C8—C1—C2—C3	59.4 (3)	C58—C51—C52—C53	59.9 (3)
C10—C1—C2—C3	−58.7 (3)	C60—C51—C52—C53	−58.6 (3)
C11—C1—C2—C3	−177.0 (2)	C61—C51—C52—C53	−176.4 (2)
C1—C2—C3—C4	60.1 (3)	C51—C52—C53—C59	−60.9 (3)
C1—C2—C3—C9	−60.8 (3)	C51—C52—C53—C54	59.9 (3)
C9—C3—C4—C5	61.4 (3)	C52—C53—C54—C55	−61.1 (3)
C2—C3—C4—C5	−59.2 (3)	C59—C53—C54—C55	58.7 (3)
C3—C4—C5—C10	58.8 (3)	C53—C54—C55—C60	61.8 (3)
C3—C4—C5—C6	−60.5 (3)	C53—C54—C55—C56	−59.0 (3)
C10—C5—C6—C7	−59.8 (3)	C60—C55—C56—C57	−59.5 (3)
C4—C5—C6—C7	60.1 (3)	C54—C55—C56—C57	60.7 (3)
C5—C6—C7—C9	−60.2 (3)	C55—C56—C57—C58	59.4 (3)
C5—C6—C7—C8	59.9 (3)	C55—C56—C57—C59	−60.7 (3)
C6—C7—C8—C1	−59.3 (3)	C56—C57—C58—C51	−60.0 (3)
C9—C7—C8—C1	61.1 (3)	C59—C57—C58—C51	59.7 (3)
C2—C1—C8—C7	−59.5 (3)	C52—C51—C58—C57	−59.1 (3)
C10—C1—C8—C7	58.3 (3)	C60—C51—C58—C57	59.8 (3)
C11—C1—C8—C7	176.5 (2)	C61—C51—C58—C57	177.6 (2)
C4—C3—C9—C7	−60.3 (3)	C52—C53—C59—C57	60.5 (3)
C2—C3—C9—C7	60.7 (3)	C54—C53—C59—C57	−59.4 (3)
C6—C7—C9—C3	59.3 (3)	C56—C57—C59—C53	59.8 (3)
C8—C7—C9—C3	−61.1 (3)	C58—C57—C59—C53	−60.2 (3)
C6—C5—C10—C1	59.7 (3)	C56—C55—C60—C51	59.9 (3)
C4—C5—C10—C1	−59.3 (3)	C54—C55—C60—C51	−61.0 (3)
C2—C1—C10—C5	58.6 (3)	C52—C51—C60—C55	59.0 (3)
C8—C1—C10—C5	−59.2 (3)	C58—C51—C60—C55	−59.6 (3)
C11—C1—C10—C5	180.0 (2)	C61—C51—C60—C55	179.5 (2)
C2—C1—C11—C12	−48.3 (3)	C52—C51—C61—C62	−72.2 (3)
C8—C1—C11—C12	73.5 (3)	C58—C51—C61—C62	49.8 (3)
C10—C1—C11—C12	−167.4 (2)	C60—C51—C61—C62	168.7 (2)
C1—C11—C12—O1	80.4 (3)	C51—C61—C62—O51	−83.7 (3)
C1—C11—C12—C13	−100.2 (3)	C51—C61—C62—C63	95.6 (3)
O1—C12—C13—C18	172.5 (2)	O51—C62—C63—C64	−1.0 (4)
C11—C12—C13—C18	−6.9 (4)	C61—C62—C63—C64	179.7 (2)
O1—C12—C13—C14	−5.2 (4)	O51—C62—C63—C68	179.4 (3)
C11—C12—C13—C14	175.4 (2)	C61—C62—C63—C68	0.2 (4)
C18—C13—C14—C15	−0.8 (4)	C64—C63—C68—C67	1.1 (4)
C12—C13—C14—C15	177.1 (2)	C62—C63—C68—C67	−179.4 (2)
C13—C14—C15—C16	0.0 (4)	C63—C68—C67—C66	−1.1 (4)
C14—C15—C16—C17	0.7 (4)	C68—C67—C66—C65	0.6 (4)
C14—C15—C16—C19	−176.5 (2)	C68—C67—C66—C69	−179.5 (2)
C15—C16—C17—C18	−0.7 (4)	C67—C66—C65—C64	−0.1 (4)
C19—C16—C17—C18	176.4 (2)	C69—C66—C65—C64	179.9 (2)
C14—C13—C18—C17	0.8 (4)	C66—C65—C64—C63	0.1 (4)
C12—C13—C18—C17	−176.9 (2)	C68—C63—C64—C65	−0.6 (4)
C16—C17—C18—C13	0.0 (4)	C62—C63—C64—C65	179.8 (2)
C20—N5—C19—C16	100.3 (3)	C70—N55—C69—C66	−99.6 (3)
C17—C16—C19—N5	146.3 (2)	C67—C66—C69—N55	−168.7 (2)
C15—C16—C19—N5	−36.6 (3)	C65—C66—C69—N55	11.3 (3)
C19—N5—C20—N1	−6.0 (3)	C69—N55—C70—N51	0.0 (3)
C19—N5—C20—C23	172.3 (2)	C69—N55—C70—C73	−177.0 (2)
C21—N1—C20—N5	177.3 (2)	C71—N51—C70—N55	−174.7 (2)
C21—N1—C20—C23	−1.1 (3)	C71—N51—C70—C73	2.3 (3)
C22—N2—C21—N1	2.1 (4)	C72—N52—C71—N51	−1.8 (4)
C22—N2—C21—Cl1	−177.54 (17)	C72—N52—C71—Cl51	178.53 (16)
C20—N1—C21—N2	−2.5 (4)	C70—N51—C71—N52	1.0 (4)
C20—N1—C21—Cl1	177.14 (17)	C70—N51—C71—Cl51	−179.33 (16)
C21—N2—C22—N4	−177.5 (2)	C71—N52—C72—N54	178.9 (2)
C21—N2—C22—C23	1.9 (3)	C71—N52—C72—C73	−0.8 (3)
C24—N4—C22—N2	179.4 (2)	C74—N54—C72—N52	179.4 (2)
C25—N4—C22—N2	2.7 (4)	C75—N54—C72—N52	−0.1 (4)
C24—N4—C22—C23	−0.2 (3)	C74—N54—C72—C73	−0.8 (2)
C25—N4—C22—C23	−176.9 (2)	C75—N54—C72—C73	179.7 (2)
N2—C22—C23—N3	−179.7 (2)	N52—C72—C73—N53	−179.3 (2)
N4—C22—C23—N3	−0.1 (3)	N54—C72—C73—N53	1.0 (3)
N2—C22—C23—C20	−5.0 (4)	N52—C72—C73—C70	3.8 (3)
N4—C22—C23—C20	174.6 (2)	N54—C72—C73—C70	−176.0 (2)
C24—N3—C23—C22	0.4 (3)	C74—N53—C73—C72	−0.7 (3)
C24—N3—C23—C20	−173.2 (3)	C74—N53—C73—C70	175.6 (2)
N5—C20—C23—C22	−174.1 (2)	N55—C70—C73—C72	172.5 (2)
N1—C20—C23—C22	4.2 (3)	N51—C70—C73—C72	−4.4 (3)
N5—C20—C23—N3	−0.8 (4)	N55—C70—C73—N53	−3.6 (4)
N1—C20—C23—N3	177.5 (2)	N51—C70—C73—N53	179.5 (2)
C23—N3—C24—N4	−0.5 (3)	C73—N53—C74—N54	0.1 (3)
C22—N4—C24—N3	0.4 (3)	C72—N54—C74—N53	0.4 (3)
C25—N4—C24—N3	177.0 (2)	C75—N54—C74—N53	179.9 (2)
C24—N4—C25—C26	111.6 (3)	C72—N54—C75—C77	76.2 (3)
C22—N4—C25—C26	−72.5 (3)	C74—N54—C75—C77	−103.2 (3)
C24—N4—C25—C27	−13.0 (4)	C72—N54—C75—C76	−158.6 (2)
C22—N4—C25—C27	162.9 (3)	C74—N54—C75—C76	22.0 (3)

### Hydrogen-bond geometry (Å, °)

**Table:**

*D*—H···*A*	*D*—H	H···*A*	*D*···*A*	*D*—H···*A*
N5—H5A···N53^i^	0.88	2.20	2.997 (3)	150
N55—H55A···N3^ii^	0.88	2.18	2.946 (3)	145
C27—H27A···Cl1^iii^	0.98	2.86	3.732 (3)	149
C54—H54B···Cl51^iv^	0.99	2.76	3.698 (3)	158

Symmetry codes: (i) −*x*+2, *y*−1/2, −*z*+3/2; (ii) −*x*+2, *y*+1/2, −*z*+3/2; (iii) −*x*+3, −*y*, −*z*+2; (iv) *x*−1, −*y*+1/2, *z*−1/2.

### Table 2 Comparative torsion angles (°) for selected 2,6,9-trisubstituted purines containing the 2-chloro 6-benzylamino and 9-isopropyl unit

**Table:**

Compound	angle	value(°)	angle	value(°)
NG38^a^	C6—N6—C9—C10	115.22 (13)	H17—C17—N9—C4	-13.38 (18)
CIBAP1^b^	C6—N6—C9—C10	178.97 (15)	H16—C16—N9—C4	-63.03 (2)
CIBAP2^c^	C6—N6—C9—C10	-117.35 (2)	H16—C16—N9—C4	30.35 (3)
CIABAP^d^	C20—N5—C19—C16	100.28 (3)	H25—C25—N4—C22	45.01 (3)
CIABAP^d^	C70—N55—C69—C66	-99.62 (3)	H75—C75—N54—C72	-40.79 (3)

Notes:  (a) Trávníček & Zatloukal (2004), where NG38 is *N*-[(2-azepan-1-yl)-9-isopropyl-9*H*-purin-6-yl]-4- methoxybenzylamine; (b) Trávníček & Popa (2007a), where CIBAP1 is 2-chloro-6-[(2,6-dimethoxybenzyl)amino]-9-isopropylpurine; (c) Trávníček & Popa (2007b), where CIBAP2 is 2-chloro-6-[(4-hydroxy-3,5-dimethoxybenzyl)amino]-9-isopropylpurine; (d) this work, where CIABAP is title compound (the structure consists of two crystallographically independent molecules).
